# Rape bee pollen outperforms camellia bee pollen in enhancing gut health and antioxidant capacity of wanxi white goose

**DOI:** 10.1016/j.psj.2025.105688

**Published:** 2025-08-19

**Authors:** Man Ren, Ji Chen, Changsheng Jiang, Fengyan Zhang, Qi Yu, Qianqian Hu, Chunfang Zhao, Ahmed H Ghonaim, Shenghe Li

**Affiliations:** a Anhui Provincial Key Laboratory of Animal Nutritional Regulation and Health, College of Animal Science, Anhui Science and Technology University, Chuzhou, 233100, China; b Local Goose Gene Bank in Anhui Province, Anhui Science and Technology University, Chuzhou 233100, China; c Anhui Engineering Technology Research Center of Pork Quality Control and Enhance, Anhui Science and Technology University, Chuzhou 233100, China; d National Key Laboratory of Agricultural Microbiology, College of Animal Sciences and Veterinary Medicine, Huazhong Agricultural University, Wuhan 430070, China; e Desert Research Center, Cairo 11435, Egypt

**Keywords:** Wanxi white goose, Rape bee pollen, Camellia bee pollen, Intestinal function, Intestinal flora

## Abstract

Bee pollen is rich in nutrients and bioactive compounds, exhibiting properties such as antioxidant effects, immune enhancement, and promotion of growth and development. However, there are limited studies on the use of bee pollen in goose breeding. This study aimed to investigate the effects of rape bee pollen (RBP) and camellia bee pollen (CBP) on production performance, intestinal morphology, digestive enzyme activity, antioxidant and immune indices, and gut microbiota in Wanxi white goose. In this study, 180 30-day-old Wanxi white goose with similar body weight were randomly divided into three groups with three replicates containing 20 goose each replicate. The control group was fed a basal diet, RBP group received the basal diet supplemented with 200 mg/kg RBP, and CBP group received the basal diet supplemented with 200 mg/kg CBP. The experiment lasted for 60 days, with sampling conducted at 60 and 90 days of age. The results revealed that RBP could significantly increase the full eviscerated rate of Wanxi white goose. Additionally, RBP and CBP significantly improved the structure of small intestine tissue, enhanced antioxidant capacity and digestive enzyme activity, and regulated the cecal microbial community structure of Wanxi white goose, with RBP demonstrating superior effects compared to CBP. Taken together, RBP and CBP significantly improved serum biochemical indices, intestinal function and the composition of intestinal flora in Wanxi white goose, with RBP demonstrating superior effects compared to CBP.

## INTRODUCTION

With the rapid development of animal husbandry, the efficiency and quality of animal breeding have garnered increasing attention. The search for and development of new green feed additives have become essential strategies for enhancing the efficiency of animal husbandry. Waterfowl production, especially that of economically important indigenous goose breeds, faces multiple challenges, including improving feed-conversion efficiency, reducing production costs, enhancing immune competence, and promoting intestinal health (Zhang et al., 2025). Against this backdrop, identifying and developing novel, eco-friendly feed additives has become a key strategy for boosting production profitability. Bee pollen, as a natural nutrient source, is rich in protein, amino acids, vitamins and minerals, as well as bioactive compounds such as flavonoids and polysaccharides. These constituents provide necessary nutritional support for animals and promote animal growth and development (Yildiz and Maskan, 2022; Sun et al., 2024). In addition, bee pollen exhibits multiple benefits, including the regulation of lipid metabolism and enhancement of antioxidant activity (Abdelnour et al., 2019; Fuliang Hu et al., 2021). A previous study has shown that bee pollen can improve the growth performance and enhance immunity of Japanese quails (Sevim, 2021). Another study found that bee pollen positively affects the growth performance and slaughter rate of broilers (Nemauluma et al., 2023). In addition, bee pollen significantly increased hepatic glutathione S-transferase (GST) and catalase (CAT) activities and decreased malondialdehyde (MDA) levels in mice fed a high-fat (HF) diet. It alleviated oxidative stress by restoring the Nrf-2–Keap-1 signaling pathway and modulated the gut microbiota by increasing the abundance of Lactobacillus and Lactococcus (Yan et al., 2021). In DSS-challenged Caco-2 intestinal epithelial cells, bee pollen upregulated the expression of antioxidant genes NQO1, Txnrd1, and Nrf2, downregulated mRNA levels of the pro-inflammatory cytokines TNF-α and IL-6, and suppressed MAPK signaling, thereby mitigating inflammatory bowel disease (Li et al., 2019). Rape bee pollen (RBP) has been found to alleviate dextran sulfate sodium (DSS)-induced colitis in mice by neutralizing IL-1β and regulating gut microbiota (Chen et al., 2019). Furthermore, camellia bee pollen (CBP) extract can reduce inflammatory cytokines in mice and regulate the structure of intestinal microbiota (Xu et al., 2021). Notably, studies on the application of bee pollen in waterfowl, especially in economically important indigenous goose breeds, are extremely scarce. Since geese differ markedly from terrestrial poultry in digestive physiology, nutritional requirements, and responses to feed additives, extrapolating findings from other species to geese lack sufficient justification. At the same time the effects of RBP and CBP on the slaughter performance and intestinal function of geese are still not fully understood. Therefore, a systematic evaluation of bee pollen’s efficacy in goose production is essential to address this research gap and to develop green feed additives specifically tailored for geese.

Wanxi white goose, an exceptional local poultry breed in Anhui province, are renowned for their tender and delicious meat, high down production and excellent quality (Yan et al., 2023; Cao et al., 2024). The meat of the Wanxi white goose is a distinctive ingredient from Anhui, characterized by its tender, firm, and elastic texture, along with its delicious taste. It is rich in high-quality protein, balanced amino acids, low fat, and high levels of unsaturated fatty acids, making it highly nutritious (Goluch and Haraf, 2023). Moreover, goose meat possesses medicinal properties; it can replenish energy, warm the stomach, and promote the production of body fluids. It is also rich in essential nutrients such as Vitamin B complex, iron, and zinc, which contribute to overall health (Yan et al., 2023). However, the breeding of Wanxi white goose faces challenges, including low feed conversion rate, high feeding cost, and decreased intestinal immunity (Zuo et al., 2024). These problems not only restrict production efficiency but may also compromise flock health and product quality.

Given the beneficial effects of bee pollen in promoting growth and enhancing immunity in animals, its distinctive properties are well suited to addressing critical challenges in Wanxi White goose production, such as low feed efficiency and weak intestinal immunity. This study incorporated 200 mg/kg CBP and RBP into the basal diet. The aim was to investigate their effects on slaughter performance, intestinal morphology, digestive enzyme activity, antioxidant and immune indices, and intestinal microflora in Wanxi white goose. This study aims to fill the research gap regarding bee-pollen application in Wanxi White goose and waterfowl in general, providing a solid scientific basis and theoretical foundation for developing novel, safe, and highly effective green feed additives specifically to this distinctive local breed.

## MATERIALS AND METHODS

### Animal Ethics Statement

All animal experiments were approved by the Animal Ethics Committee of Anhui Science and Technology University, with the protocol number 2023125. All experimental procedures were conducted in strict accordance with the “Guidelines for the Care and Use of Test Animals” of Anhui Province.

### Animals and feeding management

The geese used in this study were purchased from the Junming Wanxi white goose Breeding Base in the Yu'an District of Lu'an City. A total of 180 30-day-old healthy Wanxi white goose were randomly divided into three groups, each with three replicates containing 20 geese per replicate. The test period lasted for 60 days. Control group was fed a basal diet, while RBP group received the basal diet supplemented with 200 mg/kg of RBP, and CBP group was fed the basal diet supplemented with 200 mg/kg of CBP. RBP and CBP were purchased from Wutaishan Apiculture Co., Ltd. Experimental geese were housed in groups within a semi-open, floor-rearing system. Each pen was provided with a 5 cm layer of rice husk litter, and a 1 m² water pool was installed to allow ad libitum drinking. Throughout the entire growing period (60–90 days of age), husbandry followed the technical standards for meat-type geese. Specifically, for Wanxi White geese, the stocking density was 4–5 birds/m² from 30 to 60 days of age, and 2–3 birds/m² from 60 to 90 days of age. Natural daylight was supplemented with artificial lighting to maintain a daily photoperiod of 10–12 hours. Indoor temperature was maintained between 12°C and 22°C, with relative humidity kept at 55–60 %. The composition and nutritional components of the experimental diet are shown in Table 1.

**Table 1:** Composition and nutrient levels of the basal diet (air-dry basis) (%).

Items	30∼60 d	60∼90 d	Items	30∼60 d	60∼90 d
Diet composition (%)			Nutritional level (%) 3)		
Corn	55.84	41.55	Crude protein	17.15	15.23
Peeled soybean meal	21	19	Crude fat	2.98	2.65
Paddy	-	12	Crude fiber	4.34	6.71
Wheat bran	10	16	Calcium	0.8	0.8
Corn protein powder	5	-	Total phosphorus	0.67	0.65
Stone powder	1.05	1.3	Available phosphorus	0.43	0.36
Calcium hydrogen phosphate	0.9	0.9	Methionine	0.40	0.33
Salt	0.4	0.4	Threonine	0.80	0.77
Mineral elements premix 1)	0.05	0.05	Tryptophan	0.18	0.18
Vitamin premix 2)	0.02	0.02	L-valine	0.83	0.71
Rice husk	5	8	Isoleucine	0.70	0.58
L-lysine hydrochloride	0.05	-	Lysine	0.93	0.77
L-threonine (98 %)	0.12	0.2	Metabolic energy (MJ/kg)	11.7	10.51
DL-methionine (98 %)	0.06	0.07			
Choline chloride	0.5	0.5			
Potassium iodide	0.01	0.01			
Total	100	100			

1) Each kilogram of the basal diet contains: zinc 90 mg, manganese 80 mg, iron 80 mg, copper 20 mg, and selenium 0.3 mg. 2) Each kilogram of the basal diet contains: vitamin A 9000 IU, vitamin E 24 IU, vitamin B1 5 mg, vitamin B6 5.5 mg, vitamin D3 3000 IU, vitamin K 1.6 mg, vitamin B2 5 mg, vitamin B12 0.1 mg, folic acid 2 mg, nicotinamide 40 mg, biotin 0.05 mg, and d-pantothenic acid 15 mg. 3) All data are actual measurements.

### Sample collection

On the 30th and 60th days of the experiment, six geese from each replicate were randomly selected for sampling after ether anesthesia. The intestines (duodenum, jejunum, ileum, cecum, and colon) and the proventriculus were collected, washed with normal saline, and fixed in 4 % paraformaldehyde for tissue section and morphological observation. Simultaneously, the mucosa of the proventriculus, duodenum, jejunum, ileum and cecum were collected and stored at −80°C after being flash-frozen in liquid nitrogen for gastrointestinal function analysis. The cecal contents were collected, frozen in liquid nitrogen and stored at −80°C for microbiome sequencing analysis at end of the experiment.

For serum collection on the 30th and 60th days of the experiment, six geese were randomly selected from each replicate. After fasting for 12 h, 3–4 mL of blood was drawn from the brachial vein using a 5 mL sterile disposable syringe. The blood was immediately transferred into 5 mL clot-activator tubes without anticoagulant and allowed to clot at room temperature for 30 min. Samples were then centrifuged at 3 000 rpm for 15 min to obtain serum, which was aliquoted into 1.5 mL Eppendorf tubes and stored at –80°C until analysis of serum biochemical indicators.

### Determination of slaughter performance

On the final day of the experiment, feed was withdrawn at 08:00, while clean drinking water was provided ad libitum during a 12-hour fasting period. After fasting, six goose per replicate were randomly selected and slaughtered. Each bird was weighed to the nearest gram to record the “pre-slaughter live weight.” The geese were then restrained with both wings folded back, and exsanguination was performed by severing both carotid arteries and the jugular vein; bleeding lasted 3–5 min until complete cessation of blood flow. Subsequently, the carcasses were scalded in water at 65 ± 2°C for 60–90 s, mechanically de-feathered, and any remaining pinfeathers were removed manually. Finally, the beak sheaths, toe shells, and foot cuticles were trimmed away. The slaughter weight, semi-eviscerated weight, eviscerated weight, and subcutaneous fat thickness of the slaughtered geese were determined according to the metric statistics methods specified in NY/T 823-2020, “Poultry Production Performance Terms and Metric Statistical Methods” (Peng et al., 2023).

### Determination of serum biochemical indicators

The concentrations of cholic acid (CA), globulin (GLB), total bilirubin (TB), total protein quantitative (TP), albumin (ALB), blood sugar (GLU), uric acid (UA), total cholesterol (TC), triglyceride (TG), high-density lipoprotein (HDL), and low-density lipoprotein (LDL) in the serum were determined using kits purchased from Nanjing Jiancheng Biotechnology Co., LTD (Nanjing, China) (Ghonaim et al., 2022). Biochemical indicators were measured according to the manufacturer's instructions. The serum biochemical index determination kits are listed in Table 2.

**Table 2:** Kits used for determination of serum biochemical indices.

Kits	Catalog no.	Supplier
Cholic acid in serum assay kit (CA)	E003-1-1	Nanjing Jiancheng Bioengineer Institute, PRC
Globulin assay kit (GLB)	H547-1	
Total bilirubin assay kit (TB)	C019-1-1	
Total protein quantitative assay kit (TP)	A045-2-2	
Albumin assay kit (ALB)	A028-1-1	
Blood sugar assay kit (GLU)	H183-1-1	
Uric acid Test Kit (UA)	C012-2-1	
Total cholesterol assay kit (TC)	A111-1-1	
Triglyceride assay kit (TG)	A110-1-1	
High-density lipoprotein assay kit (HDL)	A112-1-1	
Low-density lipoprotein assay kit (LDL)	A113-1-1	

### Tissue staining

The fixed intestinal segments (duodenum, jejunum, ileum, cecum, and colon) were dehydrated, rendered transparent, and embedded in wax blocks. The wax blocks were then cut into 5 μm sections and using hematoxylin and eosin (HE) staining (Hopo et al., 2024). The intestinal morphological structure was observed using a pathological section observation system under a light microscope. Villus height, crypt depth, and muscle thickness of the intestines were measured using Image-Pro Plus 6.0 software.

### Determination of antioxidant index

The levels of reduced glutathione (GSH), total antioxidant capacity (T-AOC), total superoxide dismutase (T-SOD), and malondialdehyde (MDA) in the mucosa of duodenum, jejunum and ileum were measured using kits purchased from Nanjing Jiancheng Biotechnology Co., LTD. The antioxidant index kits are listed in Table 3.

**Table 3:** Kits for antioxidant index determination.

Kits	Catalog no.	Supplier
Reduced glutathione assay kit (GSH)	A006-2-1	Nanjing Jiancheng Bioengineer Institute, PRC
Total antioxidant capacity assay kit (T-AOC)	A015-2-1	
Total Superoxide Dismutase assay kit (T-SOD)	A001-1-2	
Malondialdehyde assay kit (MDA)	A045-2-2	

### Determination of digestive enzyme activity

The activity of digestive enzymes in the mucosa of the duodenum, jejunum, ileum and proventriculus tissue was measured using kits purchased from Nanjing Jiancheng Biotechnology Co., LTD. The digestive enzyme activity kits are listed in Table 4.

**Table 4:** Kits for digestive enzyme activity assay.

Kits	Catalog no.	Supplier
Lipase assay kit	A054-1-1	Nanjing Jiancheng Bioengineer Institute, PRC
Trypsin assay kit	A080-2-2	
α-Amylase assay kit	C016-1-1	
Maltase assay kit	A082-3-1	
Lactase assay kit	A082-1-1	
Trypsin assay kit	A080-2-2	
Pepsin assay kit	A080-1-1	

### Determination of intestinal mucosal immune function

The concentrations of immunoglobulin A (IgA), immunoglobulin G (IgG), immunoglobulin M (IgM), secretory immunoglobulin A (sIgA), interleukin-2 (IL-2), interleukin-4 (IL-4), interferon-γ (IFN-γ), and anti–tumor necrosis factor-α (TNF-α) in the mucosa of the jejunum and cecum was measured using ELISA kits according to the manufacturer's instructions. The ELISA kits used in this study are listed in Table 5.

**Table 5:** ELISA kits used in this study.

Kits	Catalog no.	Supplier
Goose immunoglobulin A (IgA) ELISA detection kit	YT-E41113	Tengyue Biotechnology Co., Ltd, Tianjin, China
Goose immunoglobulin G (IgG) ELISA detection kit	YT-E41114	
Goose immunoglobulin M (IgM) ELISA detection kit	YT-E41115	
Goose secretory immunoglobulin A (sIgA) ELISA kit	KKX-102e107	Kakaixi Biotechnology Co., Ltd., Shanghai, China
Goose interleukin-2 (IL-2) ELISA detection kit	MO-E32008	Enzyme Australia Biotechnology Co., Ltd., Shanghai, China
Goose Interleukin-4 (IL-4) ELISA Detection Kit	EKGO20	Baitai Paike Biotechnology Co., Ltd., Beijing, China
Goose Interferon-γ (IFN-γ) Kit	ml267039	Enzyme linked Biotechnology Co., Ltd., Shanghai, China
Goose Interferon-α (IFN-α) ELISA Kit	JW. GO1003	Jiwei Biotechnology Co., Ltd., Shanghai, China

### Intestinal microbiome analysis

Cecal content samples from Wanxi white goose were sent to Shenzhen Weishengtai Technology Co., Ltd. (Guangdong, China) for intestinal microbiome sequencing.

### Statistical analysis

The obtained results were expressed as the mean ± standard deviation (SD). Statistical analysis was performed using ANOVA in SPSS 20.0 software (IBM Corp., USA). Post hoc comparisons were performed using Duncan's test when ANOVA indicated significant treatment effects. In the results, different capital letters indicate highly significant differences (*P* < 0.01), different lowercase letters indicate significant differences (*P* < 0.05), and the same letter indicates non-significant differences (*P* > 0.05) within the same line.

## RESULTS

### RBP significantly increased the eviscerated rate of Wanxi white goose

The results showed that neither RBP nor CBP had a significant effect on the slaughter rate, semi-eviscerated rate, full bore rate, leg muscle rate, breast muscle rate, or subcutaneous fat of Wanxi white goose (*P* > 0.05). However, compared with the control group and the CBP group, RBP significantly increased the eviscerated rate of Wanxi white goose (*P* < 0.05) (Table 6). These findings suggest that RBP has a notable effect on improving the eviscerated rate of Wanxi white goose.

**Table 6:** Effects of RBP and CBP on slaughter performance of 90-day-old Wanxi white geese.

Items	Control group	RBP group	CBP group	*P-*value
Slaughter rate (%)	76.39±0.81	80.24±2.50	77.97±0.56	0.060
Semi-eviscerated rate (%)	71.81±0.76	75.67±2.51	71.98±0.52	0.051
Full eviscerated rate (%)	64.70±1.69^b^	70.68±2.24^a^	66.76±0.80^b^	0.013
Leg muscle rate (%)	13.29±1.90	16.03±0.26	14.32±0.80	0.079
Breast muscle rate (%)	10.98±1.48	13.29±1.90	12.22±0.87	0.209
Subcutaneous fat thickness (mm)	6.61±0.93	5.32±0.46	6.53±0.52	0.099

### RBP significantly improved the serum biochmical indices of Wanxi white goose

Compared with the control group, RBP significantly reduced the concentrations of TB and ALB in 60-day-old Wanxi white goose (*P* < 0.05), while it significantly increased the content of TP and LDL (*P* < 0.05). In contrast, compared with the control group, CBP had no significant effect on the concentrations of TB and TP (*P* > 0.05) but significantly reduced the concentration of ALB in 60-day-old Wanxi white goose (*P* < 0.05). In addition, neither RBP nor CBP had a significant effect on serum biochemical indices in 90-day-old Wanxi white goose (*P* > 0.05). These results indicate that RBP can improve the serum biochemical indices of Wanxi white goose to a certain extent.

Table 7

**Table 7:** Effects of rape bee pollen and camellia bee pollen on serum biochemical indices of Wanxi white geese aged 60/90 day.

Day age	Items	Control group	RBP group	CBP group	*P-*value
60 d	CA (mmol/L)	2.34±0.07	2.18±0.10	2.11±0.06	0.059
	GLB (g/L)	34.67±0.95	36.67±0.99	34.5 ± 0.43	0.053
	TB (g/L)	6.15±1.01^a^	3.42±0.44^b^	4.78±0.49^ab^	0.011
	TP (g/L)	41.67±1.17^b^	44.67±1.12^a^	41.67±0.49^b^	0.014
	ALB (g/L)	8.00±0.25^a^	7.00±0.36^b^	7.17±0.17^b^	0.010
	GLU (mmol/L)	9.52±0.34	9.63±0.52	9.48±0.14	0.877
	UA (µmol/L)	314.67±42.49	337.00±40.42	294.00±42.31	0.494
	TC (mmol/L)	5.51±0.30^Aa^	4.64±0.30^ABb^	4.28±0.27^Bb^	0.005
	TG (mmol/L)	0.67±0.12	0.75±0.07	0.66±0.05	0.420
	HDL (mmol/L)	2.15±0.07	2.39±0.12	2.13±0.14	0.056
	LDL (U/L)	1.48±0.21^ABb^	2.10±0.13^Aa^	1.32±0.09^Bb^	0.002
90 d	CA (mmol/L)	2.44±0.09	2.23±0.12	2.51±0.12	0.051
	GLB (g/L)	36.33±2.36	31.83±1.94	36.33±3.02	0.108
	TB (g/L)	2.75±0.31	2.47±0.27	2.83±0.24	0.313
	TP (g/L)	43.00±2.69	37.83±2.39	43.33±3.43	0.100
	ALB (g/L)	6.67±0.49	6.00±0.51	7.00±0.45	0.107
	GLU (mmol/L)	8.87±0.86	8.87±0.86	10.63±0.43	0.052
	UA (µmol/L)	410.67±45.34	327.00±34.27	437.67±55.58	0.058
	TC (mmol/L)	4.03±0.30	4.04±0.14	4.48±0.32	0.138
	TG (mmol/L)	0.87±0.12	0.86±0.10	0.87±0.13	0.993
	HDL (mmol/L)	1.83±0.15	1.59±0.10	1.87±0.15	0.088
	LDL (U/L)	1.25±0.12	1.37±0.05	1.57±0.16	0.051

Note: CA: cholic acid, GLB: globulin, TB: total bilirubin, TP: total protein, ALB: albumin, GLU: blood sugar, UA: uric acid, TC: total cholesterol, TG: triglyceride, HDL: high-density lipoprotein, LDL: low-density lipoprotein.

### RBP significantly improved the intestinal tissue structure of Wanxi white goose

In this study, the histomorphology of the intestinal tract of Wanxi white goose at 60 and 90 days of age was assessed using HE staining. Compared with the control group, RBP significantly increased the villus height and crypt depth of the ileum, as well as the villus height and villus height/crypt depth ratio (V/C value) of the cecum in 60-day-old Wanxi white goose (*P* < 0.05). Additionally, RBP significantly reduced the crypt depth of the cecum and the muscle thickness of the colon (*P* < 0.05). In addition, compared with the control group and the CBP group, RBP significantly increased the villus height of the duodenum and the V/C value of the colon in 60-day-old Wanxi white goose (*P* < 0.05 or *P* < 0.01). In contrast, CBP significantly increased the crypt depth of the duodenum, the V/C value of cecum, and the thickness of jejunum muscle layer (*P* < 0.05 or *P* < 0.01), but it also significantly reduced the crypt depth of the cecum and the muscle thickness of the colon (*P* < 0.05), compared with the control group.

**Fig 1:**
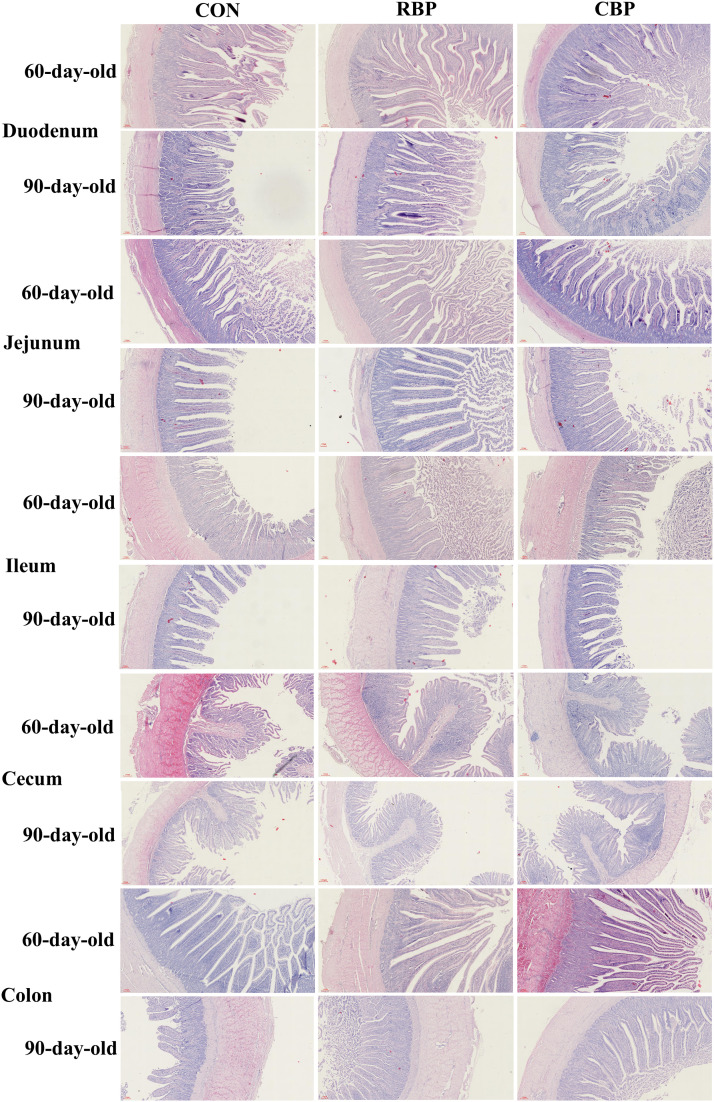
Effects of RBP and CBP on the intestinal structure of Wanxi white goose. Scale bars are 100 µm.

At 90 days of age, compared with the control group, RBP significantly increased the villus height of the duodenum and the crypt depth of the colon, while significantly reduced the crypt depth of the duodenum (*P* < 0.05). Additionally, compared with the control group and the CBP group, RBP significantly increased the V/C value of the duodenum and the villus height of the jejunum (*P* < 0.05), while significantly reduced the thickness of the ileum muscle layer (*P* < 0.05). Moreover, compared with the control group, CBP significantly reduced the muscle thickness of the jejunum in Wanxi white goose at 90 days of age (*P* < 0.05). In addition, compared with the control group and the RBP group, the CBP significantly reduced the muscle layer thickness of the jejunum and the villus height of the ileum in Wanxi white goose at 90 days of age (*P* < 0.05) (Table 8 and Fig. 1). In summary, both RBP and CBP can improve the intestinal tissue structure of Wanxi white goose, with RBP demonstrating a more pronounced effect.

**Table 8:** Effects of RBP and CBP on intestinal tissue structure of 60- and 90-day-old Wanxi white geese (µm).

Day age	Intestines	Items	Control group	RBP group	CBP group	*P-*value
60 d	Duodenum	Villi height	998.02±46.42^b^	1138.3 ± 38.89^a^	1025.8 ± 15.96^b^	0.011
		Crypt depth	325.32±15.68^b^	333.79±17.80^ab^	376.30±7.92^a^	0.013
		Muscle thickness	318.09±32.37	343.73±35.29	347.33±28.72	0.062
		Villus height/crypt depth (V/C)	3.07±0.08^ab^	3.46±0.22^a^	2.73±0.04^b^	0.013
	Jejunum	Villi height	881.43±28.01	922.57±23.46	791.82±93.98	0.080
		Crypt depth	358.73±16.54	353.00±13.28	299.82±26.38	0.059
		Muscle thickness	337.49±9.855^Bb^	416.68±26.20^ABa^	431.60±27.25^Aa^	0.005
		Villus height/crypt depth (V/C)	2.48±0.12	2.62±0.08	2.74±0.15	0.097
	Ileum	Villi height	636.63±24.30^b^	711.70±16.35^a^	659.87±13.71^ab^	0.017
		Crypt depth	275.32±26.32^b^	352.19±19.20^a^	294.39±13.81^ab^	0.010
		Muscle thickness	568.32±17.73	594.72±26.10	622.42±13.80	0.053
		Villus height/crypt depth (V/C)	2.48±0.19	2.04±0.10	2.26±0.12	0.055
	Cecum	Villi height	315.72±9.79^b^	350.06±8.26^a^	343.05±9.97^ab^	0.011
		Crypt depth	208.33±7.91^a^	167.87±9.22^bc^	176.76±12.00^b^	0.012
		Muscle thickness	469.17±11.06^ab^	447.79±11.33^b^	486.17±13.52^a^	0.022
		Villus height/crypt depth (V/C)	1.50±0.06^b^	2.10±0.09^a^	1.97±0.10^a^	0.010
	Colon	Villi height	786.49±32.32	913.13±29.56	814.01±50.33	0.056
		Crypt depth	324.72±29.92^ABa^	234.48±13.01^Bb^	364.42±18.34^Aa^	0.001
		Muscle thickness	1040.70±37.71^a^	902.20±35.46^b^	875.01±39.41^b^	0.013
		Villus height/crypt depth (V/C)	2.50±0.23^Bb^	3.96±0.33^Aa^	2.24±0.14^Bb^	<0.001
90 d	Duodenum	Villi height	924.83±18.98^b^	1062.1 ± 35.56^a^	979.26±42.53^ab^	0.015
		Crypt depth	350.34±14.11^a^	314.97±5.86^b^	329.79±10.65^ab^	0.019
		Muscle thickness	392.31±23.97	377.16±13.43	393.41±16.97	0.528
		Villus height/crypt depth (V/C)	2.60±0.13^b^	3.32±0.08^a^	3.00±0.21^b^	0.013
	Jejunum	Villi height	804.30±16.44^b^	877.34±9.122^a^	825.48±17.56^b^	0.012
		Crypt depth	347.07±18.62	369.95±14.58	347.77±13.08	0.205
		Muscle thickness	492.92±16.81^a^	489.43±22.96^a^	416.13±11.10^b^	0.013
		Villus height/crypt depth (V/C)	2.35±0.20	2.51±0.20	2.32±0.16	0.459
	Ileum	Villi height	703.75±23.55^a^	724.53±30.20^a^	624.83±25.22^b^	0.016
		Crypt depth	271.33±14.24	292.42±17.87	308.42±15.07	0.074
		Muscle thickness	526.93±29.46^a^	477.15±26.33^b^	575.16±21.29^a^	0.010
		Villus height/crypt depth (V/C)	2.56±0.12	2.47±0.09	2.26±0.15	0.058
	Cecum	Villi height	699.60±17.35	735.49±18.33	661.75±13.55	0.052
		Crypt depth	353.34±11.29^ab^	366.41±20.12^a^	320.93±10.99^b^	0.023
		Muscle thickness	487.19±22.12	540.22±22.72	489.48±16.86	0.054
		Villus height/crypt depth (V/C)	1.86±0.24	2.04±0.08	2.02±0.12	0.387
	Colon	Villi height	699.60±17.35	735.49±38.33	661.75±33.55	0.072
		Crypt depth	320.93±10.99^b^	366.41±20.12^a^	353.34±11.29^ab^	0.023
		Muscle thickness	487.19±22.12	540.22±22.72	489.48±16.86	0.054
		Villus height/crypt depth (V/C)	2.07±0.08	2.01±0.04	1.92±0.17	0.319

### RBP significantly improved the intestinal antioxidant capacity of Wanxi white goose

Compared with the control group, both CBP and RBP significantly reduced the concentration of MDA in the duodenum of Wanxi white goose at 90 days of age (*P* < 0.05). In addition, compared with the control group and the CBP group, RBP significantly increased the concentrations of T-SOD in the jejunum of 60-day-old Wanxi white goose (*P* < 0.05) and T-AOC in the ileum of 90-day-old Wanxi white goose (*P* < 0.05). Furthermore, RBP significantly increased the concentration of T-AOC in the jejunum of 90-day-old Wanxi white goose compared with control group and the CBP group (*P* < 0.05) (Table 9). These results indicate that RBP can enhance the antioxidant capacity of Wanxi white goose.

**Table 9:** Effects of RBP and CBP on antioxidant capacity in the duodenum, jejunum and ileum of Wanxi white geese.

Intestinal segment	Day age	Items	Control group	RBP group	CBP group	*P-*value
Duodenum	60 d	T-AOC (mg/mL)	0.96±0.04	0.96±0.03	0.91±0.06	0.357
		T-SOD (U/mg)	24.28±2.23	23.79±4.01	25.60±1.80	0.735
		MDA (mg/mL)	3.86±0.81	3.37±0.81	2.64±0.75	0.243
		GSH (µmol/L)	93.04±9.14	100.32±3.23	107.49±4.10	0.071
	90 d	T-AOC (mg/mL)	0.87±0.03	0.91±0.03	0.92±0.05	0.303
		T-SOD (U/mg)	32.20±1.73	29.49±1.92	33.37±3.64	0.246
		MDA (mg/mL)	7.07±2.52^a^	3.94±0.93^b^	5.84±0.78^b^	0.034
		GSH (µmol/L)	103.23±4.94	90.29±12.30	131.48±17.45	0.052
Jejunum	60 d	T-AOC (mg/mL)	0.61±0.08	0.83±0.06	0.73±0.13	0.077
		T-SOD (U/mg)	26.63±4.30^b^	40.07±2.08^a^	29.59±7.52^b^	0.042
		MDA (mg/mL)	3.01±0.93	2.69±0.69	2.19±0.68	0.472
		GSH (µmol/L)	142.57±35.01	165.56±18.32	159.99±20.89	0.556
	90 d	T-AOC (mg/mL)	0.54±0.06^b^	0.96±0.02^a^	0.70±0.06^b^	0.010
		T-SOD (U/mg)	20.37±0.71	27.14±4.46	25.41±3.89	0.117
		MDA (mg/mL)	4.24±1.09	3.85±1.36	3.64±0.96	0.816
		GSH (µmol/L)	170.93±30.38	232.40±30.90	211.92±35.29	0.137
Ileum	60 d	T-AOC (mg/mL)	0.59±0.15^b^	0.83±0.22^a^	0.60±0.33^b^	0.047
		T-SOD (mg/mL)	27.35±5.52	33.09±5.45	31.68±2.58	0.363
		MDA (mg/mL)	4.48±1.24	3.46±0.89	3.57±0.90	0.456
		GSH (µmol/L)	5.73±1.06	8.70±2.16	6.06±0.97	0.096
	90 d	T-AOC (mg/mL)	0.59±0.19	0.70±0.04	0.63±0.04	0.529
		T-SOD (mg/mL)	32.52±4.99	33.39±5.25	30.873±1.54	0.773
		MDA (mg/mL)	0.65±0.23	0.19±0.08	0.59±0.18	0.053
		GSH (µmol/L)	15.39±4.67	21.94±5.08	15.18±3.52	0.190

Note: T-AOC: total antioxidant capacity, T-SOD: total superoxide dismutase, MDA: malondialdehyde, GSH: glutathione.

### RBP significantly increased the activity of gastrointestinal digestive enzymes in Wanxi white goose

Compared with the control group and the CBP group, RBP significantly increased the pepsin activity in the proventriculus of Wanxi white goose at 90 days of age (*P* < 0.01). In the duodenum, RBP significantly increased the activity of α-amylase of Wanxi white goose at 90 days of age compared with the control group (*P* < 0.01). Both RBP and CBP significantly increased the activities of maltase and lactase (*P* < 0.05), and extremely significantly increased trypsin activity (*P* < 0.01) of Wanxi white goose at 90 days of age compared with the control group. In addition, RBP significantly increased the trypsin activity of Wanxi white goose at 90 days of age compared with the control and CBP groups (*P* < 0.05). In the jejunum: RBP significantly increased lactase activity in 60-day-old Wanxi white goose and maltase activity in 90-day-old Wanxi white goose compared with the control group (*P* < 0.05). CBP significantly increased the activity of trypsin in both 60- and 90-day-old Wanxi white goose (*P* < 0.05). In addition, RBP significantly increased the maltase activity of 60-day-old Wanxi white goose and the lipase activity of 90-day-old Wanxi white goose compared with the control and CBP group (*P* < 0.05). In the ileum: RBP and CBP significantly increased the activity of trypsin in both 60- and 90-day-old Wanxi white goose and the activity of α-amylase in 90-day-old Wanxi white goose compared with the control group (*P* < 0.05). RBP also significantly increased the activity of lactase in 90-day-old Wanxi white goose compared with control group (*P* < 0.05) (Table 10). These results suggest that RBP and CBP can improve the digestive function of Wanxi white goose, with RBP demonstrating greater effectiveness.

**Table 10:** Effects of RBP and CBP on the activities of digestive enzymes in Wanxi white geese.

Samples	Day age	Items	Control group	RBP group	CBP group	*P-*value
Proventriculus	60 d	Pepsin (U/mg)	6.61±0.29	8.11±1.38	6.70±1.32	0.259
	90 d		8.78±0.49^Bb^	13.43±1.26^Aa^	8.58±0.51^Bb^	0.001
Duodenum	60 d	Maltose (U/mg)	109.38±7.70	150.42±1.86	142.73±20.06	0.055
		Lactase (U/mg)	2.96±0.04	3.22±0.09	2.12±0.52	0.051
		Lipase (U/g)	0.69±0.01	0.82±0.02	0.65±0.13	0.076
		α-amylase (U/mg)	0.10±0.02	0.07±0.01	0.08±0.01	0.098
		Trypsin (U/mg)	1127.33±62.48^Bb^	1942.22±190.89^Aa^	1815.56±67.95^Aa^	<0.001
	90 d	Maltose (U/mg)	141.19±7.41^b^	192.83±10.67^a^	176.59±5.85^a^	0.011
		Lactase (U/mg)	1.65±0.15^b^	3.79±0.21^a^	3.52±0.33^a^	0.010
		Lipase (U/g)	1.28±0.04	1.28±0.18	0.96±0.01	0.056
		α-amylase (U/mg)	0.07±0.01^Bb^	0.11±0.01^Aa^	0.09±0.01^ABb^	0.008
		Trypsin (U/mg)	1342.67±149.34^b^	2176.89±105.81^a^	1697.33±146.81^b^	0.011
Jejunum	60 d	Maltose (U/mg)	226.02±7.34^b^	308.02±9.06^a^	254.28±13.30^b^	0.010
		Lactase (U/mg)	2.20±1.17^b^	6.23±1.22^a^	4.14±1.04^ab^	0.015
		Lipase (U/g)	1.57±0.23	1.50±0.33	0.68±0.20	0.051
		α-amylase (U/mg)	0.12±0.01	0.21±0.029	0.20±0.09	0.171
		Trypsin (U/mg)	776.22±52.79^b^	982.89±30.26^ab^	1093.56±118.42^a^	0.016
	90 d	Maltose (U/mg)	292.76±20.30^b^	478.80±41.12^a^	387.28±46.02^ab^	0.013
		Lactase (U/mg)	4.12±0.38	5.44±1.04	5.92±1.84	0.261
		Lipase (U/g)	0.81±0.14^b^	1.67±0.49^a^	0.90±0.29^b^	0.039
		α-amylase (U/mg)	0.24±0.08	0.29±0.10	0.16±0.09	0.281
		Trypsin (U/mg)	857.02±38.08^b^	1010±60.07^ab^	1054.89±29.61^a^	0.014
Ileum	60 d	Maltose (U/mg)	244.33±21.97	287.10±26.67	273.81±17.80	0.135
		Lactase (U/mg)	7.83±0.01	12.20±0.03	9.44±1.35	0.051
		Lipase (U/g)	0.95±0.16	1.15±0.27	1.10±0.28	0.603
		α-amylase (U/mg)	0.06±0.01	0.09±0.01	0.07±0.01	0.027
		Trypsin (U/mg)	1317.33±137.59^b^	2128±162.00^a^	1936±114.23^a^	0.010
	90 d	Maltose (U/mg)	306.45±24.86	411.85±41.52	330.81±25.00	0.053
		Lactase (U/mg)	5.26±1.69^b^	13.53±0.67^a^	6.40±0.78^b^	0.010
		Lipase (U/g)	1.05±0.20	1.09±0.11	1.39±0.29	0.183
		α-amylase (U/mg)	0.05±0.01^b^	0.10±0.01^a^	0.09±0.01^a^	0.012
		Trypsin (U/mg)	1625.56±238.81^b^	2693.78±157.76^a^	2364.44±201.12^a^	0.012

### RBP significantly improved the intestinal mucosa immune function of Wanxi white goose

**Fig 2:**
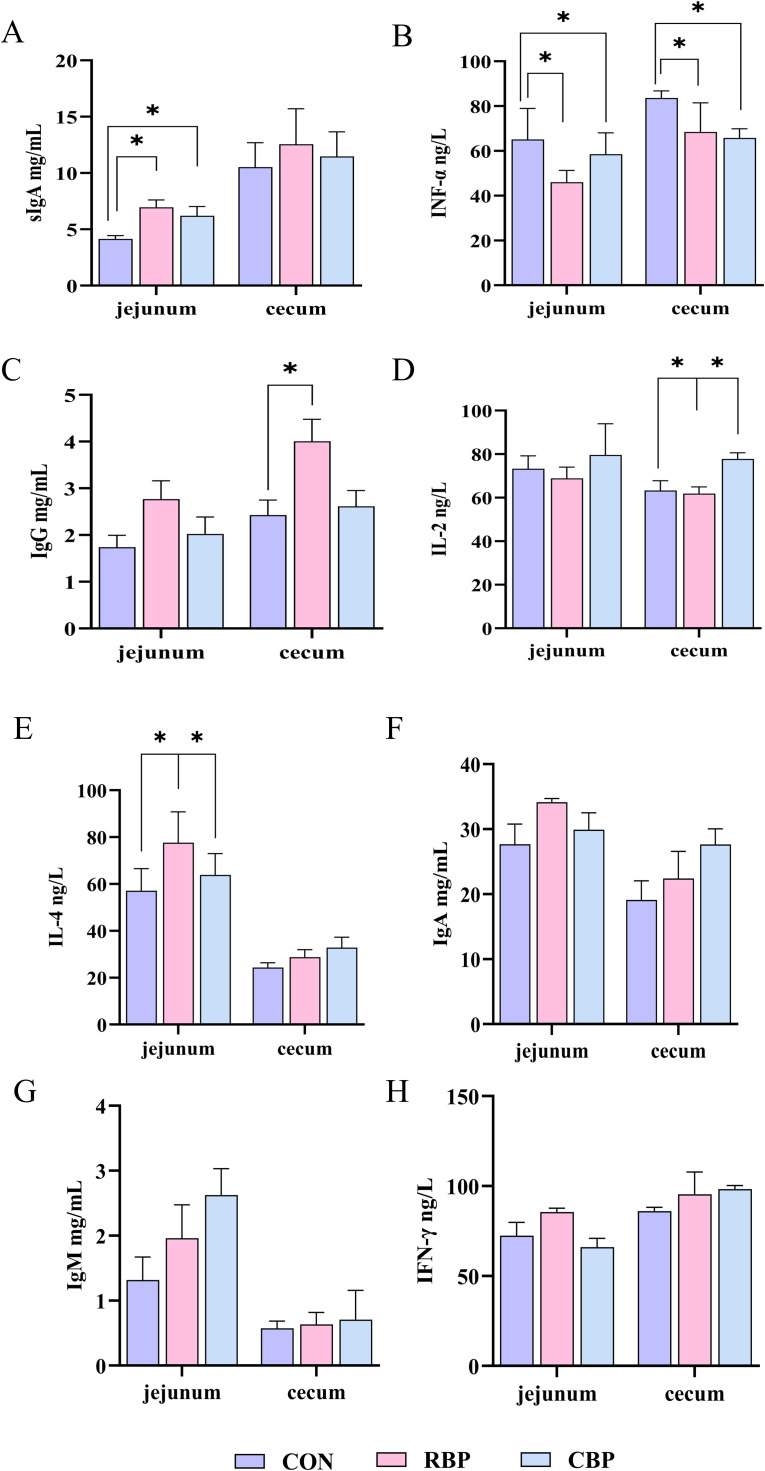
Effects of RBP and CBP on the concentration of immunoglobulins and cytokines in the intestinal mucosa of 90-day-old Wanxi white goose. (A-H) The concentrations of sIgA, INF-α, IgG, IL-2, IL-4, IgA, IgM, and IFN-γ in the jejunal and cecal mucosa of 90-day-old Wanxi white goose.

Compared with the control group, both RBP and CBP significantly increased the concentrations of secretory immunoglobulin A (sIgA) in the jejunal mucosa (Fig. 2A) and interferon-alpha (INF-α) in the jejunal and cecal mucosa (Fig. 2B) of 90-day-old Wanxi white goose. Additionally, CBP can also significantly increased the concentrations of immunoglobulin G (IgG) in the cecal mucosa of 90-day-old Wanxi white goose (*P* < 0.05) (Fig. 2C). Furthermore, compared with the control group and the CBP group, RBP significantly increased the concentrations of interleukin-2 (IL-2) and interleukin-4 (IL-4) in the cecal and jejunal mucosa of Wanxi white goose at 90 days of age (Fig. 2D, 2E) (*P* < 0.05). These results indicate that RBP and CBP can significantly improve the immune function of the intestinal mucosa of 90-day-old Wanxi white goose, with RBP demonstrating greater effectiveness.

### Analysis of cecal microbial flora

**Fig 3:**
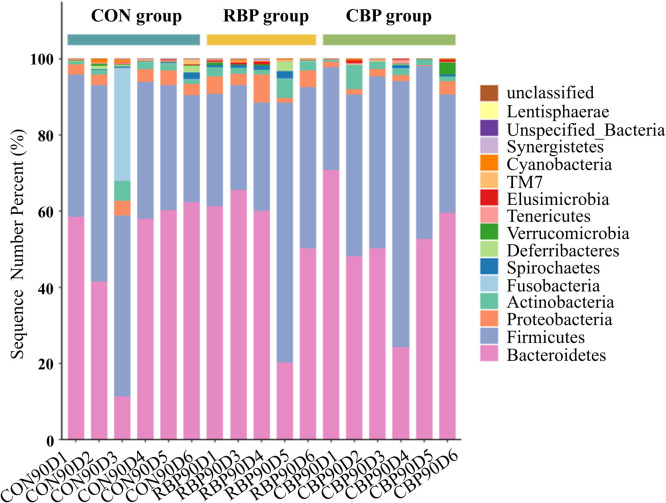
Effect of RBP and CBP on the abundance composition of cecal microflora of Wanxi white goose at the phylum level.

Species flora analysis. The composition of the cecal microbiota of Wanxi white goose at the phylum level is shown in Fig. 3. A total of 16 phyla were identified, among which the dominant phyla were Bacteroidetes, Firmicutes, Proteobacteria, Actinobacteria, Fusobacteria, Spirochaetes, Deferribacteres, and Verrucomicrobia.

As shown in Table 11, the relative abundance of bacteroidetes in the RBP group (51.57 %) and CBP group (51.04 %) was higher than that in the control group (48.71 %). The relative abundance of firmicutes in the RBP group (39.16 %) and the CBP group (43.48 %) was also higher than in control group (38.87 %). The relative abundance of proteobacteria in the RBP group (4.17 %) was higher than that in the control group (3.25 %) and the CBP group (1.68 %). The relative abundance of actinobacteria in the RBP group (2.55 %) was higher than that in the control group (2.02 %) and the CBP group (2.17 %). Conversely, the relative abundance of fusobacteria in the RBP group (0.01 %) and the CBP group (0.04 %) were lower than that in the control group (4.95 %). The relative abundance of spirochaetes in the RBP group (0.77 %) was higher than that in the control group (0.47 %) and the CBP group (0.24 %). The relative abundance of deferribacteres in the RBP group (0.57 %) was higher than that in the control group (0.53 %) and the CBP group (0.09 %). The relative abundance of verrucomicrobia in the CBP group (0.62 %) was higher than that in the control group (0.21 %) and the RBP group (0.21 %). The relative abundance of tenericutes in the RBP group (0.14 %) was lower than that in the control group (0.27 %) and the CBP group (0.30 %). The elusirnicrobia in the RBP group (0.35 %) was higher than that in the control group (0.08 %) and the CBP group (0.24 %). These results indicate that RBP can increase the abundance of Bacteroidetes, Proteobacteria and Actinobacteria in cecal contents.

**Table 11:** Species relative abundance of cecal microflora of Wanxi white geese at the phylum level (%).

Phylum	Control group	RBP group	CBP group	*P-*value
Bacteroidetes	48.71±19.70	51.57±18.21	51.04±15.36	0.979
Firmicutes	38.87±9.06	39.16±17.23	43.48±14.98	0.907
Proteobacteria	3.25±0.59	4.17±2.23	1.68±1.03	0.188
Actinobacteria	2.02±1.65	2.55±1.42	2.17±2.01	0.927
Fusobacteria	4.95±12.11	0.01±0.12	0.04±0.14	0.208
Spirochaetes	0.47±0.59	0.77±0.67	0.24±0.29	0.516
Deferribacteres	0.53±0.68	0.57±1.05	0.09±0.09	0.435
Verrucomicrobia	0.21±0.11	0.21±0.24	0.62±1.19	0.373
Tenericutes	0.27±0.31	0.14±0.14	0.30±0.33	0.705
Elusimicrobia	0.08±0.09	0.35±0.25	0.24±0.25	0.352

**Fig 4:**
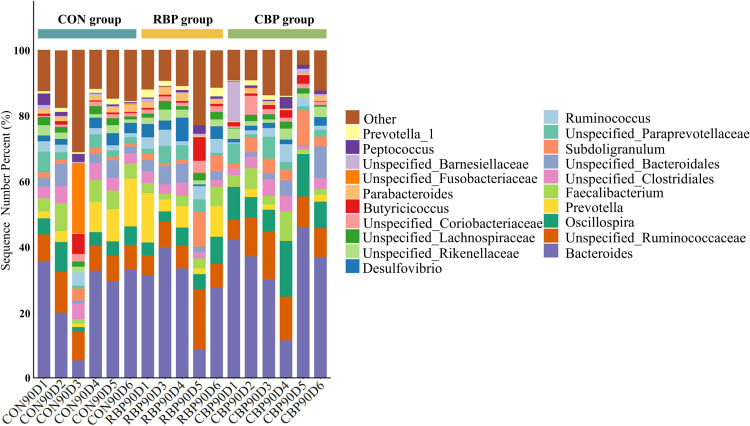
Effect of RBP and CBP on the composition of cecal microflora of Wanxi white goose at the genus level.

The composition of the cecal microbiota of Wanxi white goose at the genus level is shown in Fig. 4. A total of 21 genera were identified through species annotation. At the genus level, the top 10 dominant bacterial genera were: bacteroides, unspecified_ruminococcaceae, oscillospira, prevotella, faecalibacterium, unspecified_clostridiales, unspecified_bacteroidales, subdoligranulum and unspecified_paraprevotellaceae. The most dominant genus in the control group was bacteroides. In the control group, the most dominant genus was bacteroides. The relative abundance of bacteroides in the cecal flora of the RBP group and the CBP group was higher than that of the control group.

According to Table 12, the relative abundance of bacteroides in the RBP group (28.22 %) and the CBP group (34.10 %) was higher than that in the control group (26.05 %). The relative abundance of unspecified_ruminococcaceae in the RBP group (9.28 %) and the CBP group (10.50 %) was higher than that in the control group (8.72 %). The relative abundance of oscillospira in the RBP group (5.11 %) and the CBP group (10.25 %) was higher than that in the control group (4.96 %). The relative abundance of prevotella in the RBP group (7.23 %) was higher than that in the control group (6.65 %) and the CBP group (1.17 %). Conversely, the relative abundance of faecalibacterium in the RBP group (3.40 %) and the CBP group (3.94 %) was lower than that in the control group (5.14 %). In summary, RBP can significantly regulate the cecal microbial composition and increase the abundance of proteobacteria and other phyla, which may promote the intestinal health and physiological function of the host.

**Table 12:** Species relative abundance of cecal microflora of Wanxi white geese at the genus level (%).

Genus	Control group	RBP group	CBP group	*P-*value
Bacteroides	26.05±11.75	28.22±11.48	34.10±12.21	0.786
Unspecified_Ruminococcaceae	8.72±2.34	9.28±5.06	10.50±3.23	0.840
Oscillospira	4.96±2.50	5.11±2.03	10.25±4.10	0.125
Prevotella	6.65±5.31	7.23±5.14	1.17±0.96	0.243
Faecalibacterium	5.14±2.50	3.40±1.55	3.94±2.89	0.675
Unspecified_Clostridiales	4.35±0.90	2.89±1.03	3.28±1.52	0.357
Unspecified_Bacteroidales	3.40±2.18	3.85±1.78	2.98±3.64	0.949
Subdoligranulum	1.75±1.01	3.77±3.94	4.67±3.07	0.491
Unspecified_Paraprevotellaceae	2.86±1.77	3.30±1.91	3.12±2.48	0.967
Ruminococcus	2.42±0.99	3.50±1.39	2.25±1.74	0.535
Desulfovibrio	2.25±1.26	3.74±2.35	1.36±0.78	0.084
Unspecified_Rikenellaceae	1.90±0.64	2.20±0.81	2.22±0.93	0.864
Unspecified_Lachnospiraceae	1.83±0.01	1.84±0.01	1.35±0.01	0.050
Unspecified_Coriobacteriaceae	1.16±0.78	1.86±1.03	1.90±1.92	0.750
Butyricicoccus	1.61±2.29	1.91±2.86	1.39±0.89	0.862
Parabacteroides	1.34±0.81	1.63±0.73	0.90±0.56	0.488
Unspecified_Fusobacteriaceae	3.57±8.32	0.00±0.07	0.01±0.20	0.204
Unspecified_Barnesiellaceae	0.45±0.36	0.48±0.45	2.48±4.73	0.265
Peptococcus	1.41±1.18	0.93±0.88	1.09±1.12	0.881
Prevotella_1	0.95±0.47	1.52±1.06	0.62±0.76	0.444
Other	17.21±6.93	13.32±5.56	10.43±3.60	0.382

## DISCUSSION

RBP and CBP are rich in essential nutrients such as carbohydrates, proteins, fats, vitamins, minerals, and flavonoids (Rodríguez-Pólit et al., 2023). This study found that both RBP and CBP significantly increased the activity of digestive enzymes (lipase, trypsin, α-amylase, and others) in the small intestine of Wanxi white goose, with RBP demonstrating superior effectiveness. Specifically, RBP significantly enhanced pepsin activity in the proventriculus of Wanxi white goose at 90 days of age compared with the control and the CBP groups. In addition, RBP also significantly increased the height of intestinal villi, reduced the depth of crypts, improved the V/C value, and notably increased the eviscerated rate of Wanxi white goose. These results suggest that RBP promotes digestion and nutrient absorption by enhancing intestinal structure and increasing the activity of digestive enzymes. RBP has a higher protein content than CBP, and its rich protein and essential amino acids, such as tryptophan, provides necessary materials for synthesizing digestive enzymes (Qi et al., 2023; Sarabandi et al., 2023; Brys and Strachecka, 2024). In addition, RBP contains high levels of unsaturated fatty acids, including linolenic acid and palmitic acid (Dong et al., 2015). These unsaturated fatty acids not only support intestinal health, but also possess anti-inflammatory and lipid-regulating properties, which can mitigate intestinal inflammation and enhance nutrients absorption (Vrdoljak et al., 2022). These factors likely contribute to RBP’s ability to boost the activity of intestinal digestive enzymes and improve the slaughter rate in Wanxi white goose. A previous study has shown that bee pollen can improve the structure of colonic villi in colitis mice, increase antioxidant capacity, and regulate intestinal microflora (Chen et al., 2019). Additionally, bee pollen has been found to improve the villus height, crypt depth and V/C value in broilers, as well as enhance growth performance and immunity (Fazayeli-Rad et al., 2015). Other research found that bee pollen benefits the relative volume of intestinal epithelium and villus height, promoting the development of small intestine and intestinal function in mice (Toman et al., 2015). The reported findings not only align with our experimental results in terms of key indicators such as villus morphology, antioxidant capacity, and intestinal microbial composition, but also corroborate the broader trend that bioactive components in bee pollen can synergistically improve intestinal structure and enhance digestive enzyme activity. This convergence of evidence reinforces the biological plausibility of our results and underscores the potential applicability of RBP supplementation in poultry production systems.

RBP is rich in flavonoids such as quercetin and kaempferol, which can reduce inflammatory responses by inhibiting the production and release of inflammatory factors, while also mitigating oxidative stress damage to cells (Rodríguez-Pólit et al., 2023). In addition, flavonoids enhance immune function and improve resistance to pathogens (Denisow and Denisow-Pietrzyk, 2016; Ilie et al., 2022). These properties enable RBP to prevent and treat a variety of oxidative stress-related diseases. CBP contains a higher level of vitamin E, and RBP is abundant in vitamin E, B6, and folic acid, all of which significantly enhance the immune function of poultry (El Ghouizi et al., 2023). A previous study has reported that bee pollen can obviously increase the activities of T-AOC and T-SOD in broilers (Al-Kahtani et al., 2022). Another study also found that RBP exhibits dual effects of anti-oxidation and lipid regulation (Zhang et al., 2023). Other research indicated that compounds in RBP significantly elevate the levels of SOD and GSH in poultry, thereby improving their antioxidant capacity (Zhang et al., 2020). Moreover, bee pollen can significantly enhance the concentration of immunoglobulin (IGA, IgM) and leukocyte activity, improving the immune function of broilers (Al-Kahtani et al., 2022). It also supports intestinal barrier function, protects intestinal health and promotes the growth and development of intestinal tissue by inhibiting inflammatory responses and enhancing the expression of tight junction proteins (Zhang et al., 2024). Our study found that both RBP and CBP significantly enhanced the antioxidant capacity and immune function of the intestinal mucosa of Wanxi white goose, with RBP demonstrating superior effects. Overall, RBP has the potential to boost antioxidant capacity and enhance intestinal mucosal immune function, protecting intestinal cells from oxidative damage, and improving immune function, thereby maintaining intestinal health.

There is a symbiotic microbial community composed of bacteria and fungi in the intestines of poultry, which plays an important role in resisting pathogen invasion, promoting immune system development, and maintaining intestinal barrier function (Alam and Neish, 2018; Cheng et al., 2019)^.^ Studies have shown that bee pollen significantly affects the composition and diversity of intestinal microflora in poultry (Kačániová et al., 2013; Qiao et al., 2023). In addition, polysaccharides such as glucose and fructose found in RBP improve the health and nutritional status of poultry by increasing the relative abundance of certain beneficial bacteria during digestion and fermentation (Zhou et al., 2018). In this study, both RBP and CBP increased the relative abundance of Bacteroidetes, Firmicutes, and Proteobacteria in the cecum of Wanxi white goose. Notably, the relative abundance of Proteobacteria, Actinobacteria, Spirochaetes and Deferribacteres in the cecum was higher in the RBP group compared with the control and CBP groups. The study found that the abundance of bacteroidetes, firmicutes and proteobacteria in broiler feces increased, which helped to affect the intestinal microflora, thereby achieving optimal growth and reducing mortality, and promoting the health of broilers (Navale et al., 2024). Other study found that the addition of plant feed additives can enhance the ability of antioxidant and anti-inflammatory, and intestinal barrier function in broilers through increasing the abundance of cecal Bacteroidetes (Liu et al., 2023). At the same time, the study found that phyla such as firmicutes, bacteroidetes are stable symbiotic bacteria in the gastrointestinal tract of poultry, which can enhance intestinal digestion and absorption, support the growth of other symbiotic bacteria, maintain intestinal homeostasis, and improve feed digestibility and energy conversion efficiency (Polansky et al., 2016; Zafar and Saier, 2021). This suggests that RBP can significantly regulate the cecal microbial composition of Wanxi white goose, increasing the abundance of proteobacteria and other phyla, thus promoting the intestinal health and physiological function of the host.

## CONCLUSION

RBP and CBP significantly improved serum biochemical indices, intestinal tissue structure, intestinal antioxidant capacity, digestive enzyme activity, intestinal immune function and the composition of intestinal flora in Wanxi white goose, with RBP demonstrating superior effects compared to CBP. Therefore, RBP is a promising natural feed additive that can enhance the intestinal health of Wanxi white goose and has broad application prospects.

## Declaration of generative AI and AI-assisted technologies in the writing process

During the preparation of this work the author(s) did not use any Al and Al-assisted technologies.

## Ethics approval

All animal experiments were approved by the Animal Ethics Committee of Anhui Science and Technology University, under protocol number 2023125. All experimental procedures were carried out in strict accordance with the “Guidelines for the Care and Use of Test Animals” of Anhui Province.

## CRediT authorship contribution statement

**Man Ren:** Conceptualization, Data curation, Funding acquisition, Investigation, Methodology, Project administration, Writing – original draft, Writing – review & editing, Supervision. **Ji Chen:** Investigation, Methodology, Resources, Software, Validation, Visualization, Writing – original draft, Writing – review & editing. **Changsheng Jiang:** Data curation, Formal analysis, Funding acquisition, Methodology, Project administration, Supervision, Writing – original draft, Writing – review & editing. **Fengyan Zhang:** Conceptualization, Methodology, Resources, Software, Validation, Visualization, Writing – review & editing. **Qi Yu:** Investigation, Methodology, Visualization, Writing – review & editing. **Qianqian Hu:** Visualization, Writing – review & editing. **Chunfang Zhao:** Validation, Writing – review & editing. **Ahmed H Ghonaim:** Writing – review & editing, Validation. **Shenghe Li:** Conceptualization, Funding acquisition, Project administration, Resources, Writing – review & editing.

## Disclosures

The authors declare that they have no conflict of interest.
